# Accidental Outcomes Guide Punishment in a “Trembling Hand” Game

**DOI:** 10.1371/journal.pone.0006699

**Published:** 2009-08-26

**Authors:** Fiery Cushman, Anna Dreber, Ying Wang, Jay Costa

**Affiliations:** 1 Department of Psychology, Harvard University, Cambridge, Massachusetts, United States of America; 2 Program for Evolutionary Dynamics, Harvard University, Cambridge, Massachusetts, United States of America; 3 Department of Economics, Stockholm School of Economics, Stockholm, Sweden; Yale University, United States of America

## Abstract

How do people respond to others' accidental behaviors? Reward and punishment for an accident might depend on the actor's intentions, or instead on the unintended outcomes she brings about. Yet, existing paradigms in experimental economics do not include the possibility of accidental monetary allocations. We explore the balance of outcomes and intentions in a two-player economic game where monetary allocations are made with a “trembling hand”: that is, intentions and outcomes are sometimes mismatched. Player 1 allocates $10 between herself and Player 2 by rolling one of three dice. One die has a high probability of a selfish outcome, another has a high probability of a fair outcome, and the third has a high probability of a generous outcome. Based on Player 1's choice of die, Player 2 can infer her intentions. However, any of the three die can yield any of the three possible outcomes. Player 2 is given the opportunity to respond to Player 1's allocation by adding to or subtracting from Player 1's payoff. We find that Player 2's responses are influenced substantially by the accidental outcome of Player 1's roll of the die. Comparison to control conditions suggests that in contexts where the allocation is at least partially under the control of Player 1, Player 2 will punish Player 1 accountable for unintentional negative outcomes. In addition, Player 2's responses are influenced by Player 1's intention. However, Player 2 tends to modulate his responses substantially more for selfish intentions than for generous intentions. This novel economic game provides new insight into the psychological mechanisms underlying social preferences for fairness and retribution.

## Introduction

Jon and Matt are brothers who share two traits: poor marksmanship and a quick temper. Neither has committed a crime before, but when a rival insults their family name, both decide to take action. Jon pledges to kill the rival with a bullet to the heart, but his shot misses and the rival is unharmed. Matt just wants to spook the rival with a bullet over the shoulder, but he accidentally hits the rival's heart and kills him. In our home state of Massachusetts, Jon can expect a sentence of roughly five to ten years in prison for attempted murder with a firearm. Matt's poor marksmanship, however, earns him second degree murder—and a mandatory life sentence.

Cases like this have drawn the attention of philosophers and legal scholars [1], [2], and for good reason. They highlight a tension between two primary inputs into the process of moral judgment: the assessment of outcome versus the assessment of intent. In the case of Jon and Matt, the legal system assigns punishments in a way that depends heavily on the outcomes of their behaviors, despite their contradictory intentions. Are ordinary people's punishments of harms also strongly influenced by accidental outcomes? And, how do people respond to “accidentally generous” behavior? We investigated these questions in the context of a two-player economic game.

Games in which one player makes an allocation of money and another player rewards or punishes her by adding or subtracting money depending on that allocation have become a standard method of research in behavioral economics [3]. Yet while the differential roles of outcome and intention in moral judgment has long been a topic of psychological research [4], [5], [6], [7], [8], [9], [10], these factors have typically been confounded in economic games: the intended allocation and the actual allocation are usually identical. Put simply, there is no opportunity for error. In such studies, it cannot be known whether the response to an allocation is based on the actual allocation, the intended allocation, or an interaction between both factors.

Different economic models of responder behavior in allocator/responder games have approached this problem in different ways. According to one family of theories, we assign punishment and reward on the basis of others' intentions [11], [12]. We refer to this as the intention effect. On this model, negative responses to stingy allocations are a response to the allocator's stingy intent. According to another family of theories, we assign reward and punishment to establish an equitable distribution of resources [13], [14]. We refer to this as the distributional effect. On this model, negative responses to stingy allocations are designed to equate the final payoff between responder and allocator—thus, they depend on outcomes alone, and not on intent. Of course, the intention effect and distributional effect need not be mutually exclusive: responses in allocator/responder games may be jointly determined by both effects [15], [16].

A handful of studies have tested these two models by comparing situations in which money is allocated under full control to situations in which money is allocated by an independent mechanism, such as computerized random assignment [17], [18], [19], [20]. (Returning to the example of Matt the shooter, this is perhaps analogous to asking, “Should Matt be punished if *an independent, random event* happens to kill his rival?”). In other studies, experimenters have restricted the range of allocations available to the allocator so that the allocation is forced to be ‘stingy’ [9], [21], [22]. (This is perhaps analogous to asking, “Should Matt be punished for killing the rival if *Matt was forced* to pull the trigger?”). These experiments find that responders punish stingy allocations substantially less when the ‘allocator’ cannot control them. Such results demonstrate that the distributional effect cannot fully account for responder behavior in allocator/responder games: after all, distributions are just as unequal when made by a computer, a human, or any other mechanism. Something extra must explain the punishment of controllable allocations. It has been argued that the something extra is allocator intent; that is, the punishment of stingy allocations is largely a response to stingy intentions [9], [17], [22]. But, if it is intentions that matter, why would we punish Matt more than Jon?

Evidence from psychological research [8], [23], [24] and law suggests an important alternative account: perhaps responders failed to punish in these studies not because the allocator lacked the *intention* to make a stingy allocation, but rather because the allocator had no *control* over the allocation whatsoever. In the real world, we are required to judge individuals act with partial control over their actions, but still bring about unintended consequences—individuals like Jon and Matt, who fire their shots with a trembling hand. Why do we punish Matt severely for killing his rival, even though he intended no physical harm? Possibly, because the rival's death was caused by a behavior under Matt's partial control: the firing of the shot. We refer to this model of punishment as the “control” model: when people have at least partial control over the outcomes of their behavior, they are held accountable for those outcomes, whether specifically intended or not.

Testing the reward and punishment of monetary allocations under partial control requires an experimental paradigm where (1) an allocator can attempt to choose between different allocation amounts, (2) these choices exert partial, but imperfect control over the actual outcome of the allocation, and (3) the responder has the opportunity to respond to either the intended outcome, the actual outcome, or some combination. We therefore integrated intentions and outcomes into a single experimental condition, giving respondents the opportunity to weight the importance of each; we refer to this as a “trembling hand” game.

In this game, Player 1 allocates $10 between himself and Player 2 by choosing to roll one of three die: A, B or C. If she chooses Die A and rolls a 1, 2, 3 or 4, she receives all $10 (a “selfish” allocation); if she rolls a 5 the money is divided $5/$5 (a “fair” allocation), and if she rolls a 6, Player 2 receives all the money (a “generous” allocation). Thus, Die A has a high probability of a selfish allocation. By contrast, Die B has a high probability of a fair outcome, and Die C has a high probability of a generous outcome. When Player 1 chooses which die to roll, her intentions are transparent: she will choose the die with the highest probability of yielding the desired outcome (selfish, fair or generous). However, any die could result in any of the three possible outcomes. Thus, Player 1 has partial but imperfect control over the allocation of the $10.

Player 2 is given the opportunity to respond to Player 1's allocation by attempting to increase or decrease Player 1's payoff by any amount up to $9. Both punishments and rewards are costless to Player 2, having no effect on his payoff. Focusing particularly on cases where Player 1's intended allocation does not match the outcome of her allocation (i.e., accidents), we can assess the degree to which Player 2 assigns punishment and reward on the basis of intent, outcome, or an interaction of both factors.

In our experiment, as both players are aware, Player 2's attempt to reward or punish Player 1 is enforced by the experimenter in only 1/10 of games, selected at random. Thus, Player 1 was able to ‘get away’ with selfish allocations most of the time, without fear of punishment. This feature was introduced to convince Player 2 that ‘fair’ or ‘generous’ allocations by Player 1 were the product of genuinely prosocial motivations, rather than strategic attempts to maximize reciprocated payoffs. (To our knowledge, this feature has not been used in previous research).

What patterns of punishment and reward might we expect from the responder in the trembling hand game? The intention effect would produce responses determined exclusively by the choice of die (i.e., Player 1's intention), and not by accidental outcomes of the roll. By contrast, the distributional effect and the control effect would produce responses determined exclusively by the outcome of the roll. In order to isolate the distributional effect from the control effect, it is necessary to test a case in which allocations are made with no control. In our ‘no control’ condition, Player 1 was forced to roll a single die with equal chances of yielding all three allocations, leaving the allocation entirely to chance. The distributional model predicts that the responder will continue to add or subtract in this condition in order to equate outcomes, while the control model predicts that the responder will not reward or punish because the allocator lacks control. Thus, we can use the results of the no control condition to estimate the effect of outcomes due to the distributional model, and then test whether any additional effect of outcomes is evident in the trembling hand condition, as predicted uniquely by the control model.

We also included a ‘full control’ condition, in which Player 1 directly stipulates the amount of the allocation, without any probabilistic error. This allows us to compare the response to fully intentional allocations in the trembling hand game to the response to fully intentional allocations in a more standard allocator/responder paradigm, testing whether our results can be generalized to this large body of research.

## Methods

Research was conducted with the approval of the Committee on the Use of Human Subjects in Research at Harvard University. Written consent was obtained from every subject prior to testing.

Subjects were recruited through the online study pool at Harvard University, and comprised both students and local non-students. We report data from 30 subject pairs in each of the three conditions, totaling 180 subjects. Data from two Player 2s were excluded and replaced because matched Player 1 data was not recorded, due to experimenter error. The subjects ranged from ages 15 to 69.

All subjects were guaranteed a baseline payment of $5 for their participation in the study, supplementing the money awarded during the game. Subjects interacted anonymously with two experimenters in separate rooms. In some instances where Player 2's response could not affect Player 1's payoff, data was collected in advance from Player 1.

In the trembling hand condition, Player 2 was asked to commit to a response for each of the nine possible combinations of die chosen (Player 1's intention) and actual allocation (the outcome) before knowing which die Player 1 chose, or what she rolled. Similarly, in the control conditions Player 2 was asked to commit to a response in advance for each of the three possible outcomes. This response was described by the experimenter in terms of “additions and subtractions”, but never in terms of punishment and reward, in order to minimize demand characteristics of the game.

After Player 1's behavior was known, the experimenter then selected the appropriate response from Player 2's advance commitment (applying it with 1/10 probability and failing to apply it with 9/10 probability). This method of data collection, known as the strategy method, greatly increased our statistical power and afforded the necessary sample size of responses to low-probability events (e.g., generous intent paired with a stingy outcome). The strategy method has been widely employed in allocator-responder games, and two studies that directly compare the strategy method to sequential responses report no significant effect on responder behavior [25], [26].

Player 2's response had no effect on Player 2's monetary outcome—that is, punishment and reward were costless to Player 2. This choice served to minimize the complexity of the game: it was not necessary to introduce an additional source of variability in Player 2's payoff, to specify a ratio of cost to reward/punishment, or to explain how costs would be calculated in the 9/10 chance that reward and punishment were not imposed by the experimenter. (In pilot testing, the combination of these factors alongside the “trembling hand” technology appeared to make the game prohibitively complex for some subjects).

Subjects were provided with a written description of the rules of the game. They then participated in a structured interview with the experimenter to ensure that they fully understood the rules of the game. Player 2 used a worksheet to specify his advanced commitments to all possible moves by Player 1. After each selecting their own move, but before learning of the other player's move and the outcome of the game, both players filled out a brief questionnaire. Written instructions, response sheets, debriefing forms and a script of the structured interview are provided as supplementary information (see Supplement S1).

## Results

### The Trembling Hand Condition

**Figure 1:**
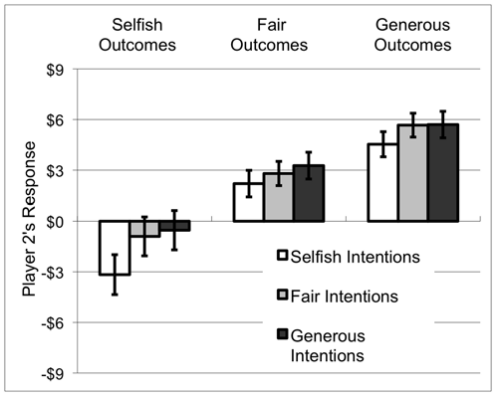
Summary of mean Player 2 responses in the trembling hand condition to each combination of intention (choice of die) and outcome (allocation amount).

In the trembling hand condition, subjects in the role of Player 2 readily responded by adding or subtracting from Player 1's payoff; not a single subject left Player 1's allocation unchanged for all nine possible moves (three possible intentions×three possible outcomes). Five subjects responded uniformly to the nine moves (each awarding $9 for every possible move). Among the remaining 25 subjects, 11 assigned punishments and rewards on the basis of the outcome alone—that is, they modified their responses according to the three different outcomes, but not according to the different intentions. No subjects assigned punishments and rewards on the basis of intent alone. 14 subjects assigned punishments and rewards on the basis of both outcome and intention. The statistical analyses below include data from all 30 subjects. Mean responses to each possible outcome of the experiment are charted in Figure 1.

In order to analyze the responses of all 30 Player 2s to the full range of 9 possible moves by Player 1, we use a linear estimator (OLS) with robust standard errors.

We include individual fixed effects since each subject responds to every possible move by Player 1 (see Methods). The dependent variable is Player 2's response, ranging from −9 to 9, where a negative number indicates punishment and a positive number reward. (Among 450 total responses, in 7 cases Player 2 indicated an addition of $10 or subtraction of $10. We changed those 7 responses to $9 and −$9, respectively, for our analysis.)

We use six independent variables to code for “stingy” outcome (β_1_), “generous” outcome (β_2_), “stingy” intent (β_3_), “generous” intent (β_4_), the interaction between stingy outcome and stingy intent (β_5_), and an interaction between generous outcome and generous intent (β_6_). Thus, our regression treated fair intent coupled with fair outcome as the baseline, with the constant term expressing the mean response to this condition, and all other coefficients expressed mean deviations from this response. The dummy for each individual Player 2 is 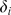. This model can be represented as:
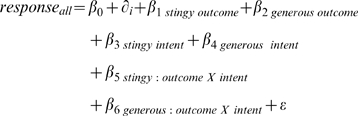



Regression analysis revealed a significant effect of stingy outcome (β_1_ = −3.82, t = −8.19, p<0.001) and of generous outcome (β_2_ = 2.60, t = 6.35, p<0.001), compared to fair outcome. There was a significant effect of selfish intention (β_3_ = −.86, t = −2.16, p<0.05) compared to fair intention, but no significant effect of generous intention (β_4_ = 0.35, t = 0.76, p = 0.45) and no significant interaction between intention and outcome (stingy, β_5_ = −1.41, t = −1.39, p = 0.17; generous β_6_ = −0.18, t = −0.22, p = 0.83). We then re-estimated the model including only significant regressors (β_1_, β_2_, β_3_), all of which remained significant at p<.01. The coefficients derived from the re-estimated model indicate that, on average, the response to stingy intentions is $1.47 less than to fair intentions, and $4.29 less for stingy outcomes compared to fair outcomes. The average response to generous outcomes is $2.54 greater than for fair outcomes.

Just over half of subjects in the position of Player 1 in the trembling hand condition chose to roll the selfish die (17/30), and the remainder chose to roll the fair die. Subjects in the role of Player 2 were asked to guess which die Player 1 would roll; 22 out of 30 guessed the selfish die, and the remainder guessed the fair die. Each Player 2 was asked to provide a written explanation of their guess regarding Player 1's choice of die, and 28/30 subjects explained their prediction in terms of the most probable outcome of the die. This suggests that nearly all subjects in the role of Player 2 understood the probabilistic weighting of the die, and the sense in which the choice of die reflected the intended allocation of Player 1.

To further safeguard against the possibility that some subjects failed to consider intentions because they did not understand the probabilistic weighting of the die, we performed a selective analysis of the 14 subjects whose individual patterns of response exhibited at least *some* sensitivity intentions—that is, whose responses were not uniform across all outcomes. There was a significant effect of stingy outcome (β_1_ = −4.18, t = −5.75, p<0.001) and of generous outcome (β_2_ = 2.44, t = 4.23, p<0.001). There was a significant effect of selfish intention (β_3_ = −1.85, t = −3.25, p<0.01), but no significant effect of generous intention (β_4_ = 0.75, t = 1.02, p = 0.31), and a marginally significant interaction between stingy intent and stingy outcome (β_5_ = −3.03, t = −1.91, p = 0.059) but no significant interaction between generous intention and generous outcome (β_6_ = −0.39, t = −0.29, p = 0.77). Thus, even this subset of subjects in the role of Player 2, who clearly assessed intentions, exhibited substantial sensitivity to accidental outcomes.

In summary, the results of the trembling hand condition reveal that Player 2 weighted the actual outcome of Player 1's allocation more heavily than Player 1's intended allocation when deciding whether to add to or subtract from Player 1's payoff. Moreover, only selfish intentions—and not generous intentions—exerted a significant influence on Player 2's behavior (comparing each to fair intentions). The response to selfish outcomes was also substantially larger than the response to generous outcomes (comparing each to fair outcomes).

### The No-control Condition

**Figure 2:**
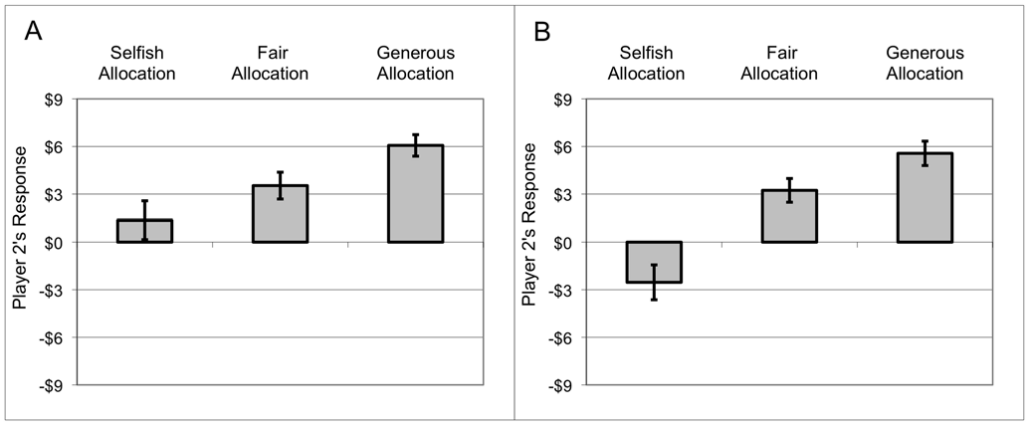
Mean Player 2 responses to “selfish”, “fair” and “generous” allocations in (A) the no control condition and (B) the full control condition.

In order to test whether Player 2's response was affected by the allocation amount in the no-control condition, we used the following OLS regression model with robust standard errors:
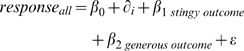
where 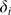 is a dummy for each individual Player 2. Mean responses are plotted in Figure 2a. Player 2′s response was significantly lower for “stingy” allocations compared to “fair” allocations: β_1_ = −2.17, t = −3.07, p<0.01. Player 2's response was also significantly higher for “generous” allocations compared to “fair” allocations: β_2_ = 2.53, t = 3.46, p<0.001. (We assume that subjects do not consider the allocations “stingy”, “fair” or “generous” when they result from uncontrollable random assignment by the roll of a single die; however, we maintain this nomenclature across all three conditions for the sake of simplicity.)

These coefficients provide an estimate of the size of the distributional effect on Player 2 responses. Can subjects' reliance on outcomes in the trembling hand condition be fully attributed to this distributional effect observed in the no-control condition? In order to provide a statistical test of this account, we conducted an OLS regression that pooled data from both the no control and trembling hand conditions. Specifically, we modeled the no control condition as a baseline, and used a dummy variable to identify the trembling hand condition. We included the three significant regressors from the analysis of the trembling hand data (stingy vs. fair outcome, generous vs. fair outcome, and stingy vs. fair intent). Finally, we included two interaction dummy variables: the interaction between condition (no control vs. trembling hand) and stingy vs. fair outcome, and the interaction between condition and generous vs. fair outcome. These interaction terms are the key regressors of interest, testing whether subjects exhibit a sensitivity to outcome in the trembling hand condition that exceeds the baseline ‘distributional’ effect observed in the no control condition. As above, we used robust standard errors and individual fixed effects. This model can be represented as:
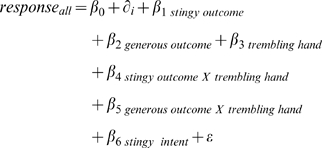



In this pooled regression, the interaction of condition with stingy compared to fair outcome was significant (β_4_ = −2.12, t = −2.75, p<0.01). However, the interaction of condition with generous vs. fair outcome was not significant (β_5_ = 0.01, t = 0.01, p = .99). (Regressors for stingy vs. fair allocation, generous vs. fair allocation, and stingy vs. fair intention all remained significant in this model). Thus, subjects were significantly more sensitive to stingy outcomes when behaviors were performed under partial control (in the trembling hand condition) compared to when behaviors were performed without control (in the no control condition). However, the response to generous outcomes performed under partial control was not significantly greater than the response to generous outcomes performed without control.

The coefficients derived from this pooled regression allow us to assign estimated effects to each of the models considered above: an intention effect, a distributional effect, and a control effect (Table 1). The intention model explains subjects' sensitivity to stingy intentions in the trembling hand condition. The distribution model explains subjects' sensitivity to both stingy and generous outcomes in the no control condition. The control model explains subjects' additional sensitivity to stingy outcomes in the trembling hand condition, compared to the no control condition.

**Table 1:** Estimated vs. Observed Effects on Player 2 Responses.

	Selfish vs. Fair	Generous vs. Fair
**Est. Intention Effect:**	−$1.47	not sig.
**Est. Distributional Effect:**	−$2.17	$2.53
**Est. Control Effect:**	−$2.12	not sig.
**Est. Full Effect (Sum):**	−$5.83	$2.53
**Observed Full Effect:**	−$5.76	$2.33

Estimated change in Player 2 response to Player 1 contrast selfish to fair allocations, and generous to fair allocations, based on three hypothesized effects: the intention effect, the distributional effect, and the control effect. Summing the hypothesized effects yields an estimate of Player 2's response to allocations made under full control (Est. Full Effect), and this can be compared to Player 2's actual response to allocations made under full control (Observed Full Effect).

### The Full Control Condition

In order to test whether Player 2's response was affected by the allocation amount in the full control condition, we used the following OLS regression model with robust standard errors:
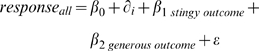
where 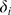 is a dummy for each individual Player 2. Player 2′s response was significantly lower for stingy allocations compared to fair allocations: β_1_ = −5.77, t = −5.14, p<0.001. Player 2's response was also significantly higher for generous allocations compared to fair allocations: β_2_ = 2.33, t = 2.42, p<0.05. Mean responses are charted in Figure 2b.

How well can the estimated coefficients derived from the trembling hand and no-control models predict Player 2's responses in the full control condition? A direct comparison of estimated versus observed effects is provided in Table 1. The total estimated effect on Player 2's response for a stingy vs. fair allocation (intention effect+distributional effect+control effect) was −$5.83, and the actual difference in mean responses to stingy vs. fair allocations in the full control condition was −$5.77. The estimated effect on Player 2's response for generous vs. fair allocation (distributional effect alone) was $2.53, and the actual difference in mean responses to generous vs. fair allocations in the full control condition was $2.33.

## Discussion

This study investigates how information about outcomes and intentions contribute to peoples' responses to selfish, fair and generous allocations of money. In particular, it explores the balance of these factors in cases where people act with partial but imperfect control over the outcome of their actions. Subjects exhibited sensitivity to both outcomes and intentions in the trembling hand condition, but outcomes tended to play a more dominant role. On average, Player 2 punished Player 1 for bad outcomes even when her intentions were good and rewarded Player 1 for good outcomes even when her intentions were bad. A strong “outcome bias” in punishment is evident in the law, and has been noted in psychological studies [10], [27], [28], [29], but recent experiments employing allocator/responder games have emphasized a relatively greater role for intentions [9], [17], [22].

By comparing responses in the trembling hand condition to responses in the no control condition, we estimated three different hypothesized effects on Player 2's response: an “intention effect” (stingy intentions are punished, generous intentions are rewarded), a “distributional effect” (Player 2 adds or subtracts money from Player 1 in order to equate their relative payoffs), and a “control effect” (stingy outcomes are punished, and generous outcomes are rewarded, but only when the outcomes are under at least partial behavioral control). Empirical estimates of these effects are summarized in Table 1. We found an intention effect for a stingy choice of die, but an even larger control effect for a stingy outcome. Neither of these effects was significant for generous outcomes compared to fair outcomes, however. Finally, we found a distributional effect comparing stingy to fair outcomes, and also comparing fair to generous outcomes.

In summary, we find that people punish accidental outcomes in allocator/responder games in a manner that cannot be explained by a pure distributional effect (i.e. aversion to inequitable outcomes). Below, we consider possible interpretations and implications of these results. First, however, we address several potential methodological concerns.

One possible explanation for the reliance on outcomes in the trembling hand condition is that subjects did not understand the probabilistic weighting of the three dice and therefore failed to consider Player 1's intentions at all. However, over two thirds of subjects in the role of Player 2 guessed that Player 1 would choose the selfish die, one third guessed that Player 1 would choose the fair die, and none guessed that Player 1 would choose the generous die. Twenty-eight of 30 subjects specifically explained their prediction about Player 1's behavior by reference to the most probable outcome of the die roll. This evidence suggests that nearly all subjects understood the probabilistic weighting of the three dice to reveal Player 1's intention to bring about a particular outcome. Furthermore, accidental outcomes played a strong role even for the subset of Player 2s whose responses clearly indicated a sensitivity to intentions.

A second possible explanation for the reliance on outcomes in the trembling hand condition is that the range of punishments available to Player 2 was restricted in the case of fair and generous allocations. When Player 1 was allocated $0, there was no money available for Player 2 to subtract from Player 1—similarly, when Player 1 was allocated $5, the maximum amount Player 2 could subtract was $5. There are reasons to doubt that this feature of the game is responsible for the dominance of outcomes in the trembling hand condition, however. First, in the trembling hand condition not a single subject subtracted the full $5 in response to a fair allocation, even when Player 1 had selfish intentions, suggesting that this value was not perceived as a limiting floor. Second, this “floor” on punishment was consistent across all conditions, and thus cannot explain the substantially different sensitivity to outcomes exhibited in the no control condition compared to the trembling hand condition. Third, in cases of selfish allocations, where the principal difference between conditions was observed, the full range of punishments and rewards was available to subjects.

Two additional methodological features of the present study are worth noting, and point towards directions for future research. First, punishment and reward were imposed by the recipient of Player 1's allocation, rather than a neutral third-party observer. Might Player 2's responses be additionally sensitive to outcomes because he experienced those outcomes himself? Possibly, but this hypothesized effect should be consistent across the trembling hand condition and no control condition—Player 1 directly experiences negative outcomes in both. Therefore, it is unlikely to explain the *additional* sensitivity to outcomes exhibited in the trembling hand condition, from which the “control effect” is inferred. Second, in our study reward and punishment were costless to Player 2. Although it is not obvious *a priori* why adding a cost to punishment and reward would selectively affect Player 2's sensitivity to intentions versus outcomes, or would selectively affect the trembling hand condition versus the no-control condition, these are important issues for further investigation.

We now turn to possible interpretations and implications of our findings, focusing on two issues in particular: (1) the distinction between the “intention effect” and “control effect” on Player 2 responses, and (2) responses to stingy versus fair behaviors, as compared to generous versus fair behaviors.

As noted above, past studies have compared experimental conditions similar to our “no control” and “full control” conditions in order to assess the importance of allocator intentions in determining punitive responses [9], [17], [18], [19], [20]. However, this experimental approach conflates two possible determinants of Player 2 responses: whether the allocation was intended by Player 1, and whether Player 1 had any control over the allocation. As we demonstrate, when these effects are differentiated the factor of controllability plays a role in determining Player 2 responses at least as large as the factor of intent. This finding accords with past psychological models implicating controllability as a key factor in retribution [8], [23], [24]. It also underscores the importance of experimental paradigms that combine intentions and outcomes probabilistically in a single game.

Why might the factor of controllability matter? We conjecture that sensitivity to behavioral control has value in repeated social interactions. That is, it may be worthwhile to ‘teach a lesson’ to an accidental harm-doer if you are likely to meet her again, but only if she can exert at least partial control over the occurrence of future harms in similar circumstances. Thus, in the context of repeated play, teaching Player 1 that stingy outcomes will be punished only makes sense if Player 1 can influence the probability of a stingy, fair or generous outcome—as was the case the “trembling hand” condition, but not in the “no control” condition. Additionally, it might be worthwhile to focus on the outcomes of another's behavior rather than their apparent intentions to protect against the possibility of deception.

Of course, neither of these considerations are justified in our experimental design: subjects interacted in an anonymous, one-shot game with no possibility of deception. However, psychological mechanisms underlying the control effect may have been shaped in contexts where repeated interactions and the possibility of deception were typical (this shaping may occur by individual learning, cultural transmission, biological evolution, or some combination). These psychological mechanisms may be automatically deployed by subjects in our experimental setting even though, strictly speaking, they are not justified.

In addition, it is notable that the intention effect and the control effect were only operative when comparing selfish to fair behaviors, and not when comparing fair to generous behaviors. Consistent with these findings, Offerman finds that punishment but not helping behavior is driven by the attribution of intentions [20]. However, Falk and colleagues find that intentions matter for both reward and punishment [17]. Responses to fair versus generous offers remains an important topic for further investigation.

Taking the results of the present study at face-value, why might subjects not have exhibited an intention effect or control effect for generous, versus fair, behavior? From a strictly quantitative, economic perspective, this distinction is hard to explain. The quantitative difference between stingy and fair behavior is just the same as between fair and generous behavior: $5 less for the allocator, and $5 more for the responder. However, psychological evidence suggests important qualitative distinctions in the way that people judge harmful versus helpful behaviors [24], [30]. Again, we conjecture that this qualitative distinction may be best understood in the context of repeated, non-anonymous interactions. When an individual (i.e. allocator) spontaneously engages in behavior categorized by the responder as “harmful”, the responder may experience a strong motivation to incentivize a change in the allocator's future behavior, for instance by punishment. But, when an individual spontaneously engages in behavior categorized by the responder as “helpful”, the responder may not experience any motivation to incentivize a change in allocator behavior—“if it ain't broke, don't fix it”. Possibly, both fair and generous allocations are generically classified by the responder as sufficiently helpful, and therefore do not motivate any incentivizing of allocator behavior.

### Conclusion

A prodigious family of studies in behavioral economics has investigated how people respond to selfish, fair and generous allocations of resources, with the consistent finding that subjects tend to diminish the payoffs of selfish allocators and increase the payoffs of generous allocators. Relatively few studies have directly investigated whether these responses are guided by a sensitivity to the actual distribution of resources or the intended distribution of resources, however. We test these factors in direct competition within a single experimental condition. The results suggest that responses to events under partial control are subject to a outcome bias: specifically, people punish ‘unlucky’ selfish allocations when performed under partial control. This effect can be dissociated from two different effects that we also confirm: an intention effect (people punish stingy intent) and a pure distributional effect (people attempt to equate final payoffs between players). These findings open new directions for research into the psychological mechanisms that underlie our social behavior.

## Supporting Information

Supplement S1 (0.04 MB DOC)
