# Supplementary material for: Accidental Outcomes Guide Punishment in a “Trembling Hand” Game
Source: PLoS One. 2009 Aug 26;4(8):e6699. doi: 10.1371/journal.pone.0006699 (PMC2726629; doi:10.1371/journal.pone.0006699)
Supplement: Supplement S1 — (0.04 MB DOC) [file pone.0006699.s001.doc]

**Supplement S1**

Below we reproduce the subject instructions and response sheets for Player 2. After each subject read through the instructions (without the labels provided in bold below) the experimenter briefly quizzed the subject on several key features of the game using the following standard script:

A lot of subjects have found these instructions confusing, so I just want to make sure that you understand everything and that we're on the same page.

First of all, this is your $5 for showing up. This money is yours no matter what, and it is separate from what you read about in the instructions. No matter what, you will leave with this $5.

Now I'd like to ask you a few questions.

(1) Can you explain to me how the $10 you read about gets divided between Player 1 and yourself?

(2) Can you explain to me how you can add or subtract money from Player 1’s payoff?

(3) If you subtract money from Player 1’s payoff, where does that money go?

An appropriately modified script was used for Player 1. At this point the Experimenter corrected any misunderstanding on the part of the subject and also answered any additional questions.

**1: Trembling Hand Condition**

Thank you for participating in this experiment.

Please read the following instructions very carefully. If anything is unclear, please ask the experimenter for clarification.

In this short experiment about decision making, you have been randomly matched with another person, "Player 1". They are in another room, and the two of you will not meet. Neither of you will ever know anything about the identity of the other.

You are guaranteed to receive a baseline payment of $5 for participating in the experiment. You can earn additional money depending on what happens in the experiment. The baseline payment and the money that you earn will be paid to you in cash immediately after the experiment is over.

There is a pool of $10 that currently belongs to both you and player 1. As a result of a random assignment, player 1 will determine how this $10 will be divided between the two of you by rolling a fair die. Player 1 has three die to choose from: A, B and C.

Die A: Roll a 1, 2, 3 or 4 and Player 1 keeps all $10.

Roll a 5 and Player 1 keeps $5 and give $5 to you.

Roll a 6 and Player 1 gives all $10 to you.

Die B: Roll a 1, 2, 3 or 4 and Player 1 keeps $5 and give $5 to you.

Roll a 5 and Player 1 keeps all $10.

Roll a 6 and Player 1 gives all $10 to you.

Die C: Roll a 1, 2, 3 or 4 and Player 1 gives all $10 to you.

Roll a 5 and Player 1 keeps $5 and give $5 to you.

Roll a 6 and Player 1 gets all $10.

You can attempt to add to or subtract from Player 1's payoff. You can add or subtract up to $9 from Player 1's payoff. It will not cost you anything to add or subtract money from player 1. Any money that is subtracted from Player 1's payoff will be returned to the experimenter. You cannot reduce Player 1's payoff below $0.

However, there is only a 1/10 chance that your attempt to add or subtract money will succeed. Chances are 9/10 that your attempt will fail, and Player 1's payoff will not be affected at all. Therefore, the probability that money will be added or subtracted from Player 1's payoff is very low. Player 1 knows that the probability of successful addition or subtraction is only 1/10.

The experimenter will use a randomized procedure to determine whether your attempt will succeed or not. Whether your attempt succeeds has nothing to do with the behavior or choices of either you or Player 1.

Please turn the page.

Before player 1 rolls the die, you must decide how you will respond to each possible outcome. All the possible outcomes are listed below. Remember, you can add or subtract up to $9 from Player 1. Circle the "+" or "-" to indicate whether you are adding to or subtracting from Player 1's payoff.

Player 1 The die Player 1 Player 1's

rolls: comes up: gets: You get: change in payoff:

Die A 1, 2 3 or 4 $10 $0 $ + / - ____

Die A 5 $5 $5 $ + / - ____

Die A 6 $0 $10 $ + / - ____

Die B 1, 2 3 or 4 $5 $5 $ + / - ____

Die B 5 $0 $10 $ + / - ____

Die B 6 $10 $0 $ + / - ____

Die C 1, 2 3 or 4 $0 $10 $ + / - ____

Die C 5 $5 $5 $ + / - ____

Die C 6 $10 $0 $ + / - ____

Player 1 will now roll the die. Money will be divided according to the roll of the die, and there is a 1/10 chance that Player 1's payoff will be modified as you have indicated.

The experiment will then be over, and you will be immediately paid in cash.

Some Final Questions

1.Which die do you think player 1 chose? Why do you think they made this choice?

2. Which die would you choose if you were player 1? Why would you make this choice?

3. What were the factors you considered when deciding whether to add or subtract money from player 1?

4. We would like to know whether you found any of the decisions about adding or subtracting money from Player 1's payoff to be particularly difficult. Please put a check mark by any decision(s) you found particularly difficult:

Player 1 rolls: The die comes up: Player 1 gets: You get: Difficulty:

Die A 1, 2 3 or 4 $10 $0 ______

Die A 5 $5 $5 ______

Die A 6 $0 $10 ______

Die B 1, 2 3 or 4 $5 $5 ______

Die B 5 $0 $10 ______

Die B 6 $10 $0 ______

Die C 1, 2 3 or 4 $0 $10 ______

Die C 5 $5 $5 ______

Die C 6 $10 $0 ______

5. What is your gender? Please circle one: Female Male

6. What is your age in years? _____

7. What is your occupation? If you are a student, what is your primary field of study?

**2: No-control Condition**

Thank you for participating in this experiment.

Please read the following instructions very carefully. If anything is unclear, please ask the experimenter for clarification.

In this short experiment about decision making, you have been randomly matched with another person, "Player 1". They are in another room, and the two of you will not meet. Neither of you will ever know anything about the identity of the other.

You are guaranteed to receive a baseline payment of $5 for participating in the experiment. You can earn additional money depending on what happens in the experiment. The baseline payment and the money that you earn will be paid to you in cash immediately after the experiment is over.

There is a pool of $10 that currently belongs to both you and player 1. As a result of a random assignment, player 1 will determine how this $10 will be divided between the two of you by rolling a fair die.

If Player 1 rolls a 1 or 2 they must keep all $10.

If Player 1 rolls a 3 or 4 they must keep $5 and give $5 to you.

If Player 1 rolls a 5 or 6 they must give you all $10.

You can attempt to add to or subtract from Player 1's payoff. You can add or subtract up to $9 from Player 1's payoff. It will not cost you anything to add or subtract money from player 1. Any money that is subtracted from Player 1's payoff will be returned to the experimenter. You cannot reduce Player 1's payoff below $0.

However, there is only a 1/10 chance that your attempt to add or subtract money will succeed. Chances are 9/10 that your attempt will fail, and Player 1's payoff will not be affected at all. Therefore, the probability that money will be added or subtracted from Player 1's payoff is very low. Player 1 knows that the probability of successful addition or subtraction is only 1/10.

The experimenter will use a randomized procedure to determine whether your attempt will succeed or not. Whether your attempt succeeds has nothing to do with the behavior or choices of either you or Player 1.

Please turn the page.

Before player 1 rolls the die, you must decide how you will respond to each possible outcome. All the possible outcomes are listed below. Remember, you can add or subtract up to $9 from Player 1. Circle the "+" or "-" to indicate whether you are adding to or subtracting from Player 1's payoff.

Player 1's

Player 1 rolls: Player 1 gets: You get: change in payoff:

1 or 2 $10 $0 $ + / - ____

3 or 4 $5 $5 $ + / - ____

5 or 6 $0 $10 $ + / - ____

Player 1 will now roll the die. Money will be divided according to the roll of the die, and there is a 1/10 chance that Player 1's payoff will be modified as you have indicated.

The experiment will then be over, and you will be immediately paid in cash.

Some Final Questions

1. What were the factors you considered when deciding whether to add or subtract money from player 1?

2. We would like to know whether you found any of the decisions about adding or subtracting money from Player 1's payoff to be particularly difficult. Please put a check mark by any decision(s) you found particularly difficult:

Player 1 rolls: Player 1 gets: You get: Difficulty:

1 or 2 $10 $0 ______

3 or 4 $5 $5 ______

5 or 6 $0 $10 ______

3. What is your gender? Please circle one: Female Male

4. What is your age in years? _____

5. What is your occupation? If you are a student, what is your primary field of study?

**3: Full-control Condition**

Thank you for participating in this experiment.

Please read the following instructions very carefully. If anything is unclear, please ask the experimenter for clarification.

In this short experiment about decision making, you have been randomly matched with another person, "Player 1". They are in another room, and the two of you will not meet. Neither of you will ever know anything about the identity of the other.

You are guaranteed to receive a baseline payment of $5 for participating in the experiment. You can earn additional money depending on what happens in the experiment. The baseline payment and the money that you earn will be paid to you in cash immediately after the experiment is over.

There is a pool of $10 that currently belongs to both you and player 1. As a result of a random assignment, player 1 will determine how this $10 will be divided between the two of you by choosing one of three options:

Option 1: Player 1 keeps all $10.

Option 2: Player 1 keeps $5 and give $5 to you.

Option 3: Player 1 gives all $10 to you.

You can attempt to add to or subtract from Player 1's payoff. You can add or subtract up to $9 from Player 1's payoff. It will not cost you anything to add or subtract money from player 1. Any money that is subtracted from Player 1's payoff will be returned to the experimenter. You cannot reduce Player 1's payoff below $0.

However, there is only a 1/10 chance that your attempt to add or subtract money will succeed. Chances are 9/10 that your attempt will fail, and Player 1's payoff will not be affected at all. Therefore, the probability that money will be added or subtracted from Player 1's payoff is very low. Player 1 knows that the probability of successful addition or subtraction is only 1/10.

The experimenter will use a randomized procedure to determine whether your attempt will succeed or not. Whether your attempt succeeds has nothing to do with the behavior or choices of either you or Player 1.

Please turn the page.

Before player 1 rolls the die, you must decide how you will respond to each possible outcome. All the possible outcomes are listed below. Remember, you add or subtract up to $9 from Player 1. Circle the "+" or "-" to indicate whether you are adding to or subtracting from Player 1's payoff.

Player 1 Player 1's

chooses: Player 1 gets: You get: change in payoff:

Option 1 $10 $0 $ + / - ____

Option 2 $5 $5 $ + / - ____

Option 3 $0 $10 $ + / - ____

Player 1 will now choose an option. Money will be divided according to option Player 1 chooses, and there is a 1/10 chance that Player 1's payoff will be modified as you have indicated.

The experiment will then be over, and you will be immediately paid in cash.

Some Final Questions

1.Which option do you think player 1 chose? Why do you think they made this choice?

2. Which option would you choose if you were player 1? Why would you make this choice?

3. What were the factors you considered when deciding whether to add or subtract money from player 1?

4. We would like to know whether you found any of the decisions about adding or subtracting money from Player 1's payoff to be particularly difficult. Please put a check mark by any decision(s) you found particularly difficult:

Player 1

chooses: Player 1 gets: You get: Difficulty:

Option 1 $10 $0 ______

Option 2 $5 $5 ______

Option 3 $0 $10 ______

5. What is your gender? Please circle one: Female Male

6. What is your age in years? _____

7. What is your occupation? If you are a student, what is your primary field of study?
